# Supplementary material for: Altered Muscle–Brain Connectivity During Left and Right Biceps Brachii Isometric Contraction Following Sleep Deprivation: Insights from PLV and PDC
Source: Sensors (Basel). 2025 Mar 28;25(7):2162. doi: 10.3390/s25072162 (PMC11991489; doi:10.3390/s25072162)
Supplement: Supplementary file 1 [file sensors-25-02162-s001.zip › Supplemental File 1. Sleep duration, RPE scores, Duration of biceps isometric contractions and MVC values of participants..docx]

**Supplementary file 1**

**Table 1:** Sleep duration and RPE scores of participants.

| Participants No. | Good sleep | |  | Poor sleep | |
| --- | --- | --- | --- | --- | --- |
|  | Sleep time (min) | RPE level |  | Sleep time (min) | RPE level |
| 1 | 450 | 6 |  | 180 | 16 |
| 2 | 460 | 7 |  | 185 | 15 |
| 3 | 445 | 7 |  | 230 | 15 |
| 4 | 430 | 9 |  | 129 | 17 |
| 5 | 442 | 6 |  | 137 | 16 |
| 6 | 483 | 7 |  | 233 | 15 |
| 7 | 465 | 6 |  | 125 | 16 |
| 8 | 430 | 7 |  | 145 | 17 |
| 9 | 475 | 7 |  | 220 | 15 |
| 10 | 486 | 6 |  | 175 | 15 |
| 11 | 445 | 8 |  | 209 | 15 |
| 12 | 455 | 7 |  | 193 | 17 |
| 13 | 455 | 9 |  | 230 | 17 |
| 14 | 440 | 7 |  | 236 | 16 |
| 15 | 444 | 9 |  | 210 | 15 |
| 16 | 450 | 9 |  | 229 | 17 |
| 17 | 445 | 6 |  | 166 | 17 |
| 18 | 425 | 7 |  | 165 | 16 |
| 19 | 435 | 6 |  | 162 | 18 |
| 20 | 446 | 7 |  | 196 | 17 |
| 21 | 440 | 6 |  | 127 | 18 |
| 22 | 450 | 6 |  | 177 | 16 |
| 23 | 460 | 7 |  | 210 | 15 |
| 24 | 470 | 7 |  | 205 | 17 |
| 25 | 430 | 6 |  | 156 | 18 |
| 26 | 485 | 9 |  | 142 | 19 |
| 27 | 430 | 7 |  | 215 | 15 |
| 28 | 440 | 6 |  | 230 | 17 |
| 29 | 435 | 7 |  | 175 | 17 |
| 30 | 456 | 7 |  | 236 | 16 |
| 31 | 458 | 8 |  | 190 | 15 |
| 32 | 467 | 7 |  | 237 | 16 |
| 33 | 464 | 7 |  | 161 | 18 |
| 34 | 473 | 6 |  | 180 | 15 |
| 35 | 438 | 8 |  | 140 | 16 |

Note: Sleep time recording instrument: GT9X-BT (ActiLife Inc., US); Criteria of poor sleep: sleep time < 240 min; RPE ≥ level 15; Criteria of good sleep: sleep time ≥ 420 min (7 h); RPE < level 10. Rating of Perceived Exertion (RPE) (Gunnar Borg, 1998).

**Table 2**: Duration of biceps isometric contractions of participants.

|  | Left | |  | Right | |
| --- | --- | --- | --- | --- | --- |
|  | good sleep | poor sleep |  | good sleep | poor sleep |
| 1 | 427.33 | 330.71 |  | 365.03 | 332.48 |
| 2 | 367.25 | 367.42 |  | 332.88 | 331 |
| 3 | 375.69 | 321.65 |  | 310.93 | 303.75 |
| 4 | 314.47 | 330.88 |  | 364.91 | 311.56 |
| 5 | 383.15 | 378.82 |  | 313.21 | 316.06 |
| 6 | 338.52 | 309.91 |  | 371.7 | 336.98 |
| 7 | 363.89 | 323.53 |  | 366.97 | 305.52 |
| 8 | 365.94 | 325.3 |  | 366.86 | 337.61 |
| 9 | 365.21 | 333.51 |  | 379.49 | 331 |
| 10 | 337.36 | 313.1 |  | 376.2 | 333.45 |
| 11 | 369.53 | 306.32 |  | 392.39 | 320.68 |
| 12 | 383.21 | 364.17 |  | 413.28 | 316.52 |
| 13 | 366.52 | 383.26 |  | 360.75 | 337.9 |
| 14 | 331.13 | 352.97 |  | 311.02 | 365.12 |
| 15 | 313.38 | 336.56 |  | 374.7 | 317.07 |
| 16 | 377.05 | 318.19 |  | 367.19 | 317.97 |
| 17 | 363.83 | 309.29 |  | 388.02 | 374.86 |
| 18 | 337.79 | 310.63 |  | 358.94 | 351.12 |
| 19 | 367.51 | 393.24 |  | 364.56 | 310.69 |
| 20 | 413.34 | 376.3 |  | 374.64 | 375.78 |
| 21 | 381.3 | 369.34 |  | 380.97 | 367.95 |
| 22 | 363.94 | 372.82 |  | 369.46 | 374.22 |
| 23 | 392.43 | 365.17 |  | 378.74 | 332.05 |
| 24 | 394.17 | 318.48 |  | 383.44 | 372.24 |
| 25 | 368.94 | 289.48 |  | 366.85 | 321.44 |
| 26 | 382.39 | 339.07 |  | 364.3 | 342.55 |
| 27 | 426.18 | 333.5 |  | 303.75 | 370.16 |
| 28 | 382.28 | 266.58 |  | 334.43 | 321.15 |
| 29 | 366.56 | 264.24 |  | 331.99 | 332.76 |
| 30 | 384.08 | 270.18 |  | 390.4 | 332.48 |
| 31 | 377.29 | 382.62 |  | 390.4 | 295.92 |
| 32 | 374.97 | 364.68 |  | 332.86 | 316.74 |
| 33 | 367.14 | 309.78 |  | 346.49 | 307.8 |
| 34 | 312.85 | 306.6 |  | 308.68 | 309.24 |
| 35 | 326.31 | 334.14 |  | 326.6 | 313.14 |

**Table 3**: The MVC values of the subject's left and right biceps (1-100Hz).

|  | Left | |  | Right | |
| --- | --- | --- | --- | --- | --- |
|  | iEMG | RMS |  | iEMG | RMS |
| 1 | 3278.10 | 1340.93 |  | 2029.76 | 656.52 |
| 2 | 4683.84 | 1265.85 |  | 1994.50 | 755.76 |
| 3 | 3787.58 | 1408.81 |  | 2176.43 | 821.17 |
| 4 | 3472.63 | 1367.95 |  | 1780.23 | 666.47 |
| 5 | 2939.36 | 1055.29 |  | 2423.66 | 781.15 |
| 6 | 3761.58 | 882.36 |  | 2362.96 | 687.91 |
| 7 | 3478.70 | 1496.48 |  | 1705.73 | 811.48 |
| 8 | 3698.49 | 1644.83 |  | 2668.03 | 810.30 |
| 9 | 5104.65 | 1183.73 |  | 2864.60 | 818.55 |
| 10 | 3181.91 | 1092.78 |  | 2751.50 | 879.44 |
| 11 | 4092.56 | 1083.79 |  | 1411.16 | 872.57 |
| 12 | 3199.80 | 1333.77 |  | 2608.75 | 840.48 |
| 13 | 2552.45 | 904.94 |  | 2138.10 | 955.04 |
| 14 | 2618.25 | 818.60 |  | 3702.88 | 960.07 |
| 15 | 4094.11 | 1578.74 |  | 1782.57 | 642.48 |
| 16 | 4781.82 | 1382.42 |  | 2170.22 | 915.86 |
| 17 | 3415.24 | 1088.13 |  | 2554.71 | 880.70 |
| 18 | 4147.58 | 1516.87 |  | 1667.19 | 718.39 |
| 19 | 3690.59 | 1219.57 |  | 1773.95 | 723.95 |
| 20 | 4852.31 | 1451.27 |  | 2330.85 | 822.75 |
| 21 | 4907.63 | 1410.23 |  | 2243.00 | 1019.21 |
| 22 | 3151.70 | 961.04 |  | 2243.00 | 807.33 |
| 23 | 2720.76 | 769.93 |  | 1870.99 | 756.49 |
| 24 | 3200.78 | 1011.09 |  | 2947.86 | 840.42 |
| 25 | 3787.58 | 1118.49 |  | 1737.40 | 821.47 |
| 26 | 3773.56 | 1435.80 |  | 2141.90 | 618.05 |
| 27 | 4706.20 | 1277.49 |  | 2391.25 | 771.44 |
| 28 | 3796.29 | 1043.75 |  | 2698.45 | 896.23 |
| 29 | 5411.26 | 1303.19 |  | 2865.20 | 774.15 |
| 30 | 4025.23 | 1288.80 |  | 1821.47 | 658.34 |
| 31 | 4691.53 | 1489.14 |  | 1953.97 | 671.06 |
| 32 | 1966.59 | 911.19 |  | 1823.43 | 1004.21 |
| 33 | 4723.45 | 1141.48 |  | 2580.88 | 843.72 |
| 34 | 3352.87 | 1495.77 |  | 1609.35 | 921.83 |
| 35 | 3518.44 | 1425.98 |  | 2679.20 | 831.41 |
