# Supplementary material for: Altered Muscle–Brain Connectivity During Left and Right Biceps Brachii Isometric Contraction Following Sleep Deprivation: Insights from PLV and PDC
Source: Sensors (Basel). 2025 Mar 28;25(7):2162. doi: 10.3390/s25072162 (PMC11991489; doi:10.3390/s25072162)
Supplement: Supplementary file 1 [file sensors-25-02162-s001.zip › Supplemental File 2. Motor sensation related brain areas and corresponding channels..docx]

**Supplemental File 2.**

Motor sensation related brain areas and corresponding channels.

| **Related motor cortex** | **Brodmann area** | **Channels** |
| --- | --- | --- |
| Premotor cortex | 8 | F3, F11, FZ, F2, F4 |
| Supplementary motor cortex | 6 | FC3, FC1, FCZ, FC2, FC4 |
| Primary somatosensory motor cortex | 1, 2, 3, 4 | C3, C4 |
| Secondary sensorimotor cortex | 5 | CP1, CP2 |
