# Supplementary material for: Altered Muscle–Brain Connectivity During Left and Right Biceps Brachii Isometric Contraction Following Sleep Deprivation: Insights from PLV and PDC
Source: Sensors (Basel). 2025 Mar 28;25(7):2162. doi: 10.3390/s25072162 (PMC11991489; doi:10.3390/s25072162)
Supplement: Supplementary file 1 [file sensors-25-02162-s001.zip › Supplemental File 3. Comparison of global attributes among ROI based on PLV matrix data..docx]

**Supplemental File 3.**

Comparison of global attributes among ROI based on PLV matrix data.

| Frequency Band | Index | Factors **(sleep × states)** | Resting state | Left-bicep contraction | Right-bicep contraction | *F* | *df* | *p* | *ɳ^2^* |
| --- | --- | --- | --- | --- | --- | --- | --- | --- | --- |
| θ | sigma | Good sleep | 0.104 ± 0.020 | 0.104 ± 0.022 | 0.112 ± 0.020 |  |  |  |  |
|  |  | Poor sleep | 0.110 ± 0.018 | 0.104 ± 0.022 | 0.113 ± 0.028 |  |  |  |  |
|  |  | Interaction effect |  |  |  | 0.385 | (2,68) | 0.682 | 0.011 |
|  |  | Main effect in Sleep |  |  |  | 0.827 | (1,34) | 0.370 | 0.024 |
|  |  | Main effect in States |  |  |  | 2.325 | (2,68) | 0.106 | 0.064 |
|  | E_g_ | Good sleep | 0.0206 ± 0.0028 | 0.0193 ± 0.0030 | 0.0191 ± 0.0030 |  |  |  |  |
|  |  | Poor sleep | 0.0200 ± 0.0030 | 0.0197 ± 0.0033 | 0.0193 ± 0.0030 |  |  |  |  |
|  |  | Interaction effect |  |  |  | 0.668 | (2,68) | 0.516 | 0.019 |
|  |  | Main effect in Sleep |  |  |  | 0.001 | (1,34) | 0.982 | 0.000 |
|  |  | Main effect in States |  |  |  | 2.318 | (2,68) | 0.106 | 0.064 |
|  | E_loc_ | Good sleep | 0.0310 ± 0.0041 | 0.0326 ± 0.0055 | 0.0338 ± 0.0053 |  |  |  |  |
|  |  | Poor sleep | 0.0335 ± 0.0051 | 0.0320 ± 0.0050 | 0.0334 ± 0.0054 |  |  |  |  |
|  |  | Interaction effect |  |  |  | 2.032 | (2,68) | 0.139 | 0.056 |
|  |  | Main effect in Sleep |  |  |  | 0.463 | (1,34) | 0.501 | 0.013 |
|  |  | Main effect in States |  |  |  | 1.807 | (2,68) | 0.172 | 0.050 |
| α | sigma | Good sleep | 0.143 ± 0.025 | 0.146 ± 0.030 | 0.162 ± 0.016 ^##^ |  |  |  |  |
|  |  | Poor sleep | 0.154 ± 0.024 | 0.145 ± 0.029 | 0.157 ± 0.030 |  |  |  |  |
|  |  | Interaction effect |  |  |  | 1.570 | (2,68) | 0.215 | 0.044 |
|  |  | Main effect in Sleep |  |  |  | 0.246 | (1,34) | 0.623 | 0.007 |
|  |  | Main effect in States |  |  |  | 5.473 | (2,68) | **0.006** | 0.139 |
|  | E_g_ | Good sleep | 0.0286 ± 0.0032 | 0.0264 ± 0.0034 ^#^ | 0.0238 ± 0.0029 ^###&&^ |  |  |  |  |
|  |  | Poor sleep | 0.0279 ± 0.0039 | 0.0273 ± 0.0045 | 0.0270 ± 0.0040 ^***^ |  |  |  |  |
|  |  | Interaction effect |  |  |  | 5.146 | (2,68) | **0.008** | 0.131 |
|  |  | Simple effect in Sleep (level 1) |  |  |  | 17.716 | (2,33) | **0.000** | 0.518 |
|  |  | Simple effect in States (level 3) |  |  |  | 13.949 | (1,34) | **0.001** | 0.291 |
|  | E_loc_ | Good sleep | 0.0433 ± 0.0054 | 0.0456 ± 0.0074 | 0.0496 ± 0.0050 ^###^ |  |  |  |  |
|  |  | Poor sleep | 0.0467 ± 0.0069 ^*^ | 0.0450 ± 0.0069 | 0.0469 ± 0.0074 |  |  |  |  |
|  |  | Interaction effect |  |  |  | 3.495 | (2,68) | **0.036** | 0.093 |
|  |  | Simple effect in Sleep (level 1) |  |  |  | 10.567 | (2,33) | **0.000** | 0.390 |
|  |  | Simple effect in States (level 1) |  |  |  | 5.996 | (1,34) | **0.020** | 0.150 |
| β | sigma | Good sleep | 0.389 ± 0.053 | 0.372± 0.061 | 0.386 ± 0.049 |  |  |  |  |
|  |  | Poor sleep | 0.398 ± 0.048 | 0.371 ± 0.073 | 0.393± 0.063 |  |  |  |  |
|  |  | Interaction effect |  |  |  | 0.128 | (2,68) | 0.880 | 0.004 |
|  |  | Main effect in Sleep |  |  |  | 0.322 | (1,34) | 0.574 | 0.009 |
|  |  | Main effect in States |  |  |  | 3.788 | (2,68) | **0.028** | 0.100 |
|  | E_g_ | Good sleep | 0.0769 ± 0.0064 | 0.0732 ± 0.0083 | 0.0721 ± 0.0068 |  |  |  |  |
|  |  | Poor sleep | 0.0747 ± 0.0068 | 0.0727 ± 0.0073 | 0.0747 ± 0.0076 |  |  |  |  |
|  |  | Interaction effect |  |  |  | 1.911 | (2,68) | 0.156 | 0.053 |
|  |  | Main effect in Sleep |  |  |  | 0.000 | (1,34) | 0.984 | 0.000 |
|  |  | Main effect in States |  |  |  | 2.905 | (2,68) | 0.062 | 0.079 |
|  | E_loc_ | Good sleep | 0.123 ± 0.009 | 0.125 ± 0.011 | 0.127 ± 0.009 |  |  |  |  |
|  |  | Poor sleep | 0.126 ± 0.010 | 0.124 ± 0.013 | 0.126 ± 0.010 |  |  |  |  |
|  |  | Interaction effect |  |  |  | 0.755 | (2,68) | 0.474 | 0.022 |
|  |  | Main effect in Sleep |  |  |  | 0.132 | (1,34) | 0.719 | 0.004 |
|  |  | Main effect in States |  |  |  | 0.692 | (2,68) | 0.504 | 0.020 |

Note: *Vs.* good sleep, ^*^: *p* < 0.05, ^***^: *p* < 0.001. *Vs.* resting state, ^#^: *p* < 0.05, ^##^: *p* < 0.01, ^###^: *p* < 0.001. *Vs.* left-bicep, ^&&^: *p* < 0.01.
