# Supplementary material for: Altered Muscle–Brain Connectivity During Left and Right Biceps Brachii Isometric Contraction Following Sleep Deprivation: Insights from PLV and PDC
Source: Sensors (Basel). 2025 Mar 28;25(7):2162. doi: 10.3390/s25072162 (PMC11991489; doi:10.3390/s25072162)
Supplement: Supplementary file 1 [file sensors-25-02162-s001.zip › Supplemental File 4. PDC value on muscle and primary motor sensory cortex after sleep deprivation..docx]

**Supplemental File 4.**

PDC value on muscle and primary motor sensory cortex after sleep deprivation.

| Frequency band | Direction of causality | Left biceps contraction | | | |  | Right biceps contraction | | | |
| --- | --- | --- | --- | --- | --- | --- | --- | --- | --- | --- |
|  |  | Good sleep (mean) | Poor sleep (mean) | *p* | trend |  | Good sleep (mean) | Poor sleep (mean) | *p* | trend |
| β ^1^ (13~20Hz) | C3 → Biceps | 0.5756 | 0.5725 | *0.999* | - |  | 0.5750 | 0.5794 | *0.546* | - |
|  | C4 → Biceps | 0.5756 | 0.5725 | *0.999* | - |  | 0.5750 | 0.5794 | *0.364* | - |
|  | C3 → C4 | 0.5814 | 0.5815 | *0.999* | - |  | 0.5799 | 0.5784 | *0.395* | - |
|  | C4 → C3 | 0.5738 | 0.5769 | *0.999* | - |  | 0.5767 | 0.5734 | *0.581* | - |
|  | Biceps → C3 | 0.5738 | 0.5769 | *0.999* | - |  | 0.5767 | 0.5734 | *0.465* | - |
|  | Biceps → C4 | 0.5814 | 0.5814 | *0.997* | - |  | 0.5788 | 0.5784 | *0.444* | - |
| β ^2^ (20~30Hz) | C3 → Biceps | 0.5675 | 0.5820 | *0.131* | - |  | 0.5768 | 0.5798 | *0.999* | - |
|  | C4 → Biceps | 0.5674 | 0.5819 | *0.087* | - |  | 0.5768 | 0.5798 | *0.608* | - |
|  | C3 → C4 | 0.5843 | 0.5680 | *0.116* | - |  | 0.5789 | 0.5783 | *0.677* | - |
|  | C4 → C3 | 0.5772 | 0.5795 | *0.226* | - |  | 0.5761 | 0.5733 | *0.486* | - |
|  | Biceps → C3 | 0.5772 | 0.5795 | *0.254* | - |  | 0.5761 | 0.5733 | *0.607* | - |
|  | Biceps → C4 | 0.5843 | 0.5680 | *0.173* | - |  | 0.5789 | 0.5783 | *0.592* | - |
| γ ^1^ (30~60Hz) | C3 → Biceps | 0.5671 | 0.5807 | 0.012 | ↑^*^ |  | 0.5794 | 0.5902 | 0.201 | - |
|  | C4 → Biceps | 0.5671 | 0.5808 | **0.014** | **↑^*^** |  | 0.5794 | 0.5903 | 0.300 | - |
|  | C3 → C4 | 0.5849 | 0.5724 | 0.009 | ↓^**^ |  | 0.5780 | 0.5676 | 0.179 | - |
|  | C4 → C3 | 0.5767 | 0.5778 | 0.034 | ↑^*^ |  | 0.5741 | 0.5641 | 0.998 | - |
|  | Biceps → C3 | 0.5765 | 0.5778 | 0.037 | ↑^*^ |  | 0.5741 | 0.5642 | 0.554 | - |
|  | Biceps → C4 | 0.5847 | 0.5724 | **0.013** | **↓^*^** |  | 0.5780 | 0.5677 | 0.158 | - |
| γ ^2^ (60~100Hz) | C3 → Biceps | 0.5766 | 0.5689 | 0.114 | - |  | 0.5751 | 0.5723 | 0.276 | - |
|  | C4 → Biceps | 0.5766 | 0.5689 | 0.340 | - |  | 0.5751 | 0.5724 | 0.215 | - |
|  | C3 → C4 | 0.5772 | 0.5810 | 0.113 | - |  | 0.5767 | 0.5934 | 0.062 | - |
|  | C4 → C3 | 0.5759 | 0.5799 | 0.152 | - |  | 0.5792 | 0.5634 | 0.019 | ↓^*^ |
|  | Biceps → C3 | 0.5758 | 0.5798 | 0.181 | - |  | 0.5793 | 0.5633 | 0.021 | ↓^*^ |
|  | Biceps → C4 | 0.5771 | 0.5810 | 0.127 | - |  | 0.5768 | 0.5632 | **0.033** | **↓^*^** |

Note: C3 and C4 channels represent the discharge signals of the left and right primary motor sensory cortex, respectively.

↑: up, ↓: down, vs. good sleep, *: *p* < 0.05, **: *p* < 0.01, - : not passed FDR correction.
