# Supplementary material for: Altered Muscle–Brain Connectivity During Left and Right Biceps Brachii Isometric Contraction Following Sleep Deprivation: Insights from PLV and PDC
Source: Sensors (Basel). 2025 Mar 28;25(7):2162. doi: 10.3390/s25072162 (PMC11991489; doi:10.3390/s25072162)
Supplement: Supplementary file 1 [file sensors-25-02162-s001.zip › Supplemental File 1. Sleep duration, RPE scores, Duration of biceps isometric contractions and MVC values of participants.pdf]

Supplementary file 1

Table S1: Sleep duration and RPE scores of participants.

Participants No.	Good sleep		Poor sleep	
	Sleep time (min)	RPE level	Sleep time (min)	RPE level
1	450	6	180	16
2	460	7	185	15
3	445	7	230	15
4	430	9	129	17
5	442	6	137	16
6	483	7	233	15
7	465	6	125	16
8	430	7	145	17
9	475	7	220	15
10	486	6	175	15
11	445	8	209	15
12	455	7	193	17
13	455	9	230	17
14	440	7	236	16
15	444	9	210	15
16	450	9	229	17
17	445	6	166	17
18	425	7	165	16
19	435	6	162	18
20	446	7	196	17
21	440	6	127	18
22	450	6	177	16
23	460	7	210	15
24	470	7	205	17
25	430	6	156	18
26	485	9	142	19
27	430	7	215	15
28	440	6	230	17
29	435	7	175	17
30	456	7	236	16
31	458	8	190	15
32	467	7	237	16
33	464	7	161	18
34	473	6	180	15
35	438	8	140	16

Note: Sleep time recording instrument: GT9X-BT (ActiLife Inc., US); Criteria of poor sleep: sleep time < 240 min; RPE ≥ level 15; Criteria of good sleep: sleep time ≥ 420 min (7 h); RPE < level 10. Rating of Perceived Exertion (RPE) (Gunnar Borg, 1998).

Table S2: Duration of biceps isometric contractions of participants.

	Left		Right	
	good sleep	poor sleep	good sleep	poor sleep
1	427.33	330.71	365.03	332.48
2	367.25	367.42	332.88	331
3	375.69	321.65	310.93	303.75
4	314.47	330.88	364.91	311.56
5	383.15	378.82	313.21	316.06
6	338.52	309.91	371.7	336.98
7	363.89	323.53	366.97	305.52
8	365.94	325.3	366.86	337.61
9	365.21	333.51	379.49	331
10	337.36	313.1	376.2	333.45
11	369.53	306.32	392.39	320.68
12	383.21	364.17	413.28	316.52
13	366.52	383.26	360.75	337.9
14	331.13	352.97	311.02	365.12
15	313.38	336.56	374.7	317.07
16	377.05	318.19	367.19	317.97
17	363.83	309.29	388.02	374.86
18	337.79	310.63	358.94	351.12
19	367.51	393.24	364.56	310.69
20	413.34	376.3	374.64	375.78
21	381.3	369.34	380.97	367.95
22	363.94	372.82	369.46	374.22
23	392.43	365.17	378.74	332.05
24	394.17	318.48	383.44	372.24
25	368.94	289.48	366.85	321.44
26	382.39	339.07	364.3	342.55
27	426.18	333.5	303.75	370.16
28	382.28	266.58	334.43	321.15
29	366.56	264.24	331.99	332.76
30	384.08	270.18	390.4	332.48
31	377.29	382.62	390.4	295.92
32	374.97	364.68	332.86	316.74
33	367.14	309.78	346.49	307.8
34	312.85	306.6	308.68	309.24
35	326.31	334.14	326.6	313.14

Table S3: The MVC values of the subject's left and right biceps (1-100Hz).

	Left		Right	
	iEMG	RMS	iEMG	RMS
1	3278.10	1340.93	2029.76	656.52
2	4683.84	1265.85	1994.50	755.76
3	3787.58	1408.81	2176.43	821.17
4	3472.63	1367.95	1780.23	666.47
5	2939.36	1055.29	2423.66	781.15
6	3761.58	882.36	2362.96	687.91
7	3478.70	1496.48	1705.73	811.48
8	3698.49	1644.83	2668.03	810.30
9	5104.65	1183.73	2864.60	818.55
10	3181.91	1092.78	2751.50	879.44
11	4092.56	1083.79	1411.16	872.57
12	3199.80	1333.77	2608.75	840.48
13	2552.45	904.94	2138.10	955.04
14	2618.25	818.60	3702.88	960.07
15	4094.11	1578.74	1782.57	642.48
16	4781.82	1382.42	2170.22	915.86
17	3415.24	1088.13	2554.71	880.70
18	4147.58	1516.87	1667.19	718.39
19	3690.59	1219.57	1773.95	723.95
20	4852.31	1451.27	2330.85	822.75
21	4907.63	1410.23	2243.00	1019.21
22	3151.70	961.04	2243.00	807.33
23	2720.76	769.93	1870.99	756.49
24	3200.78	1011.09	2947.86	840.42
25	3787.58	1118.49	1737.40	821.47
26	3773.56	1435.80	2141.90	618.05
27	4706.20	1277.49	2391.25	771.44
28	3796.29	1043.75	2698.45	896.23
29	5411.26	1303.19	2865.20	774.15
30	4025.23	1288.80	1821.47	658.34
31	4691.53	1489.14	1953.97	671.06
32	1966.59	911.19	1823.43	1004.21
33	4723.45	1141.48	2580.88	843.72
34	3352.87	1495.77	1609.35	921.83
35	3518.44	1425.98	2679.20	831.41
