# Supplementary material for: Altered Muscle–Brain Connectivity During Left and Right Biceps Brachii Isometric Contraction Following Sleep Deprivation: Insights from PLV and PDC
Source: Sensors (Basel). 2025 Mar 28;25(7):2162. doi: 10.3390/s25072162 (PMC11991489; doi:10.3390/s25072162)
Supplement: Supplementary file 1 [file sensors-25-02162-s001.zip › Supplemental File 2. Motor sensation related brain areas and corresponding channels.pdf]

Supplemental File 2.

Motor sensation related brain areas and corresponding channels.

**Related motor cortex**	**Brodmann area**	**Channels**
Premotor cortex	8	F3, F11, FZ, F2, F4
Supplementary motor cortex	6	FC3, FC1, FCZ, FC2, FC4
Primary somatosensory motor cortex	1, 2, 3, 4	C3, C4
Secondary sensorimotor cortex	5	CP1, CP2
