# Supplementary material for: Altered Muscle–Brain Connectivity During Left and Right Biceps Brachii Isometric Contraction Following Sleep Deprivation: Insights from PLV and PDC
Source: Sensors (Basel). 2025 Mar 28;25(7):2162. doi: 10.3390/s25072162 (PMC11991489; doi:10.3390/s25072162)
Supplement: Supplementary file 1 [file sensors-25-02162-s001.zip › Supplemental File 3. Comparison of global attributes among ROI based on PLV matrix data.pdf]

Supplemental File 3.

Comparison of global attributes among ROI based on PLV matrix data.

Frequency Band	Index	Factors **(sleep × states)**	Resting state	Left-bicep contraction	Right-bicep contraction	*F*	*df*	*p*	η^2
θ	sigma	Good sleep	0.104 ± 0.020	0.104 ± 0.022	0.112 ± 0.020				
		Poor sleep	0.110 ± 0.018	0.104 ± 0.022	0.113 ± 0.028				
		Interaction effect				0.385	(2,68)	0.682	0.011
		Main effect in Sleep				0.827	(1,34)	0.370	0.024
		Main effect in States				2.325	(2,68)	0.106	0.064
	E_g	Good sleep	0.0206 ± 0.0028	0.0193 ± 0.0030	0.0191 ± 0.0030				
		Poor sleep	0.0200 ± 0.0030	0.0197 ± 0.0033	0.0193 ± 0.0030				
		Interaction effect				0.668	(2,68)	0.516	0.019
		Main effect in Sleep				0.001	(1,34)	0.982	0.000
		Main effect in States				2.318	(2,68)	0.106	0.064
	E_{loc}	Good sleep	0.0310 ± 0.0041	0.0326 ± 0.0055	0.0338 ± 0.0053				
		Poor sleep	0.0335 ± 0.0051	0.0320 ± 0.0050	0.0334 ± 0.0054				
		Interaction effect				2.032	(2,68)	0.139	0.056
		Main effect in Sleep				0.463	(1,34)	0.501	0.013
		Main effect in States				1.807	(2,68)	0.172	0.050
α	sigma	Good sleep	0.143 ± 0.025	0.146 ± 0.030	0.162 ± 0.016 $^{\#\#}$				
		Poor sleep	0.154 ± 0.024	0.145 ± 0.029	0.157 ± 0.030				
		Interaction effect				1.570	(2,68)	0.215	0.044
		Main effect in Sleep				0.246	(1,34)	0.623	0.007
		Main effect in States				5.473	(2,68)	**0.006**	0.139
	E_g	Good sleep	0.0286 ± 0.0032	0.0264 ± 0.0034 $^{\#}$	0.0238 ± 0.0029 $^{\#\#\#\&\&}$				
		Poor sleep	0.0279 ± 0.0039	0.0273 ± 0.0045	0.0270 ± 0.0040 ***				

		Interaction effect				5.146	(2,68)	**0.008**	0.131
		Simple effect in Sleep (level 1)				17.716	(2,33)	**0.000**	0.518
		Simple effect in States (level 3)				13.949	(1,34)	**0.001**	0.291
	E_{loc}	Good sleep	0.0433 ± 0.0054	0.0456 ± 0.0074	0.0496 ± 0.0050 ###				
		Poor sleep	0.0467 ± 0.0069 *	0.0450 ± 0.0069	0.0469 ± 0.0074				
		Interaction effect				3.495	(2,68)	**0.036**	0.093
		Simple effect in Sleep (level 1)				10.567	(2,33)	**0.000**	0.390
		Simple effect in States (level 1)				5.996	(1,34)	**0.020**	0.150
β	sigma	Good sleep	0.389 ± 0.053	0.372± 0.061	0.386 ± 0.049				
		Poor sleep	0.398 ± 0.048	0.371 ± 0.073	0.393± 0.063				
		Interaction effect				0.128	(2,68)	0.880	0.004
		Main effect in Sleep				0.322	(1,34)	0.574	0.009
		Main effect in States				3.788	(2,68)	**0.028**	0.100
	E_g	Good sleep	0.0769 ± 0.0064	0.0732 ± 0.0083	0.0721 ± 0.0068				
		Poor sleep	0.0747 ± 0.0068	0.0727 ± 0.0073	0.0747 ± 0.0076				
		Interaction effect				1.911	(2,68)	0.156	0.053
		Main effect in Sleep				0.000	(1,34)	0.984	0.000
		Main effect in States				2.905	(2,68)	0.062	0.079
	E_{loc}	Good sleep	0.123 ± 0.009	0.125 ± 0.011	0.127 ± 0.009				
		Poor sleep	0.126 ± 0.010	0.124 ± 0.013	0.126 ± 0.010				
		Interaction effect				0.755	(2,68)	0.474	0.022
		Main effect in Sleep				0.132	(1,34)	0.719	0.004
		Main effect in States				0.692	(2,68)	0.504	0.020

Note: *Vs.* good sleep, *: $p < 0.05$, ***: $p < 0.001$. *Vs.* resting state, #: $p < 0.05$, ##: $p < 0.01$, ###: $p < 0.001$. *Vs.* left-bicep, &&: $p < 0.01$.
