# Supplementary material for: Altered Muscle–Brain Connectivity During Left and Right Biceps Brachii Isometric Contraction Following Sleep Deprivation: Insights from PLV and PDC
Source: Sensors (Basel). 2025 Mar 28;25(7):2162. doi: 10.3390/s25072162 (PMC11991489; doi:10.3390/s25072162)
Supplement: Supplementary file 1 [file sensors-25-02162-s001.zip › Supplemental File 4. PDC value on muscle and primary motor sensory cortex after sleep deprivation.pdf]

Supplemental File 4.

PDC value on muscle and primary motor sensory cortex after sleep deprivation.

Frequency band	Direction of causality	Left biceps contraction				Right biceps contraction			
		Good sleep (mean)	Poor sleep (mean)	*p*	trend	Good sleep (mean)	Poor sleep (mean)	*p*	trend
β 1 (13~20Hz)	C3 → Biceps	0.5756	0.5725	*0.999*	-	0.5750	0.5794	*0.546*	-
	C4 → Biceps	0.5756	0.5725	*0.999*	-	0.5750	0.5794	*0.364*	-
	C3 → C4	0.5814	0.5815	*0.999*	-	0.5799	0.5784	*0.395*	-
	C4 → C3	0.5738	0.5769	*0.999*	-	0.5767	0.5734	*0.581*	-
	Biceps → C3	0.5738	0.5769	*0.999*	-	0.5767	0.5734	*0.465*	-
	Biceps → C4	0.5814	0.5814	*0.997*	-	0.5788	0.5784	*0.444*	-
β 2 (20~30Hz)	C3 → Biceps	0.5675	0.5820	*0.131*	-	0.5768	0.5798	*0.999*	-
	C4 → Biceps	0.5674	0.5819	*0.087*	-	0.5768	0.5798	*0.608*	-
	C3 → C4	0.5843	0.5680	*0.116*	-	0.5789	0.5783	*0.677*	-
	C4 → C3	0.5772	0.5795	*0.226*	-	0.5761	0.5733	*0.486*	-
	Biceps → C3	0.5772	0.5795	*0.254*	-	0.5761	0.5733	*0.607*	-
	Biceps → C4	0.5843	0.5680	*0.173*	-	0.5789	0.5783	*0.592*	-
γ 1 (30~60Hz)	C3 → Biceps	0.5671	0.5807	0.012	↑*	0.5794	0.5902	0.201	-
	C4 → Biceps	0.5671	0.5808	**0.014**	↑*	0.5794	0.5903	0.300	-
	C3 → C4	0.5849	0.5724	0.009	↓**	0.5780	0.5676	0.179	-
	C4 → C3	0.5767	0.5778	0.034	↑*	0.5741	0.5641	0.998	-
	Biceps → C3	0.5765	0.5778	0.037	↑*	0.5741	0.5642	0.554	-
	Biceps → C4	0.5847	0.5724	**0.013**	↓*	0.5780	0.5677	0.158	-
γ 2 (60~100Hz)	C3 → Biceps	0.5766	0.5689	0.114	-	0.5751	0.5723	0.276	-
	C4 → Biceps	0.5766	0.5689	0.340	-	0.5751	0.5724	0.215	-
	C3 → C4	0.5772	0.5810	0.113	-	0.5767	0.5934	0.062	-
	C4 → C3	0.5759	0.5799	0.152	-	0.5792	0.5634	0.019	↓*
	Biceps → C3	0.5758	0.5798	0.181	-	0.5793	0.5633	0.021	↓*
	Biceps → C4	0.5771	0.5810	0.127	-	0.5768	0.5632	**0.033**	↓*

Note: C3 and C4 channels represent the discharge signals of the left and right primary motor sensory cortex, respectively.

↑: up, ↓: down, vs. good sleep, *: $p < 0.05$, **: $p < 0.01$, - : not passed FDR correction.
